# Progressive Injury in Chronic Multiple Sclerosis Lesions Is Gender-Specific: A DTI Study

**DOI:** 10.1371/journal.pone.0149245

**Published:** 2016-02-22

**Authors:** Alexander Klistorner, Chenyu Wang, Con Yiannikas, Stuart L. Graham, John Parratt, Michael H. Barnett

**Affiliations:** 1 Department of Ophthalmology, Save Sight Institute, University of Sydney, Sydney, Australia; 2 Australian School of Advanced Medicine, Macquarie University, Sydney, NSW, Australia; 3 Sydney Neuroimaging Analysis Centre, Sydney, NSW, Australia; 4 Brain and Mind Centre, University of Sydney, Sydney, NSW, Australia; 5 North Shore Hospital, Sydney, NSW, Australia; Institute Biomedical Research August Pi Sunyer (IDIBAPS) - Hospital Clinic of Barcelona, SPAIN

## Abstract

**Objective:**

To evaluate the longitudinal integrity of white matter tracts in patients with relapsing remitting multiple sclerosis (RRMS) as determined by changes in diffusivity indices of lesional and non-lesional white matter in the optic radiation over 12 months.

**Methods:**

The optic radiation (OR) was identified in sixty RRMS patients using probabilistic tractography. MS lesions were segmented on FLAIR T2 images and a lesion mask was intersected with the co-registered OR. Lesions within the OR were identified in 39 patients. Voxel-based analysis of axial diffusivity (AD) and radial diffusivity (RD) within OR lesions and non-lesional normal appearing white matter (NAWM) was performed at baseline and 12 months in 34 patients (five patients excluded due to new OR lesions).

**Results:**

Both RD and AD demonstrated much higher values within the lesions compared with non-lesional NAWM. There was a significant (p<0.001) increase of lesional AD and RD during the follow-up period. This increase, however, was driven almost entirely by the male cohort, in which a significantly greater change in both AD (M-2.7%, F-0.9%) and RD (M-4.6%, F-0.7%) was observed during the follow-up period. Non-lesional NAWM also demonstrated an increase in both AD and RD, albeit on a much lesser scale (1.0% and 0.6% respectively). In contradistinction to lesions, the diffusivity change in non-lesional NAWM was similar between sexes.

**Conclusions:**

The evolution of AD and RD in chronic MS lesions over 12 months suggests ongoing inflammatory demyelinating activity accompanied by axonal loss. In addition, our findings are consistent with the recently observed trend of more rapid clinical progression in males and establish a potential *in vivo* biomarker of gender dichotomy by demonstrating a significantly faster rate of microstructural change in the chronic lesions of male patients with MS.

## Introduction

Multiple sclerosis (MS) is a complex disease of the CNS, characterized by inflammation, demyelination, neuro-axonal loss and gliosis. Inflammatory demyelinating lesions are a hallmark of the disease. However, neuro-axonal loss is believed to underpin the progressive disability that characterizes MS. While acute lesional damage is a major cause of axonal loss in MS, chronic inflammation at the edge of lesions (“lesion burning”) and loss of trophic support from myelin in the depths of the chronic lesion together with diffuse inflammatory changes in normal appearing white matter (NAWM) may contribute to progressive axonal damage[1][2]^.^

Diffusion tensor imaging (DTI) is a non-conventional MRI technique based on diffusion of water molecules in brain tissue[3]. It is sensitive to the microstructural organization of white matter tracts and has been suggested as a new promising tool in MS that provides greater pathological specificity than conventional MRI, helping, therefore, to elucidate disease pathogenesis and monitor therapeutic efficacy[4].

Earlier studies have linked axial (i.e. parallel to axonal direction) diffusivity to axonal loss; and radial (i.e. perpendicular to axonal direction) diffusivity with myelin content[5][6]. However, this paradigm is now considered an over-simplification, particularly since diffusivity indices can potentially (and sometimes paradoxically) be affected by crossing, branching, merging or kissing fibers among other factors[7].

We have recently demonstrated that tract-specific analysis partially alleviates this problem by examining highly coherent fibers of a single (optic radiation) pathway[8]. Therefore, to provide insight into longitudinal white matter tract integrity in MS, in the current study we evaluated changes in diffusivity indices of lesional and non-lesional white matter of the optic radiation during 12 months follow-up and its potential relation to gender, age, duration of disease and lesion load.

## Methods

### Subjects

All procedures followed the tenets of the Declaration of Helsinki and written informed consent was obtained from all participants. Study was approved by Sydney University Human Ethics board.

In this prospective study sixty consecutive RRMS patients were enrolled. Patients with any other systemic or ocular diseases were excluded. A history of optic neuritis (ON) was not an exclusion criteria, however, none of the patients had ON within 6 months of baseline assessment. MRI was performed at study enrolment and 12 months later. Since new lesions may significantly impact on diffusivity (particularly of the NAWM), patients who had gadolinium (Gad)-enhanced lesions at baseline or developed new lesions (Gad or FLAIR) during follow-up were excluded from analysis.

### MRI protocol

The following sequences were acquired using a 3T GE Discovery MR750 scanner (GE Medical Systems, Milwaukee, WI):

1. Pre- and post contrast (gadolinium) Sagittal 3D T1: GE BRAVO sequence, FOV 256mm, Slice thickness 1mm, TE 2.7ms, TR 7.2ms, Flip angle 12°, Pixel spacing 1mm. Acquisition Matrix (Freq.× Phase) is 256×256, which results in 1mm isotropic acquisition voxel size. The reconstruction matrix is 256x256.
2. FLAIR CUBE; GE CUBE T2 FLAIR sequence, FOV 240mm, Slice thickness 1.2mm, Acquisition Matrix (Freq.× Phase) 256×244, TE 163ms, TR 8000ms, Flip angle 90°, Pixel spacing 0.47 mm. The reconstruction matrix is 512x512.
3. Whole brain diffusion-weighted images using a spin echo, 64 directions, FOV 256 mm, Acquisition Matrix (Freq.× Phase) 128×128, slice thickness 2mm, TE 83ms, TR 8325ms, b-value = 1000 and number of b = 0 acquisitions = 2. The reconstruction matrix is 256x256.

### Tractography

Probabilistic tractography was used to reconstruct OR fibers as previously described in detail elsewhere[9]. Briefly, after eddy-current correction and motion compensation, DTI and FLAIR T2 images were co-registered to the high resolution T1 structural image. To reduce the effect of EPI susceptibility distortion, non-linear registration-based correction was used for DTI co-registration. Identification of two regions of interest (ROI), the lateral geniculate nucleus (LGN) and the occipital cortex, facilitated the implementation of probabilistic tractography of the optic radiation. To identify the LGN, which is nearly invisible on structural T1-weighted images, optic tract fibers were followed from the optic chiasm using deterministic tractography (a 10 mm ROI placed on the optic chiasm was used to seed the deterministic algorithm). The position of the LGN was inferred by the termination of optic tract fibers, at which point a circular ROI (diameter 7 mm) was placed. An occipital cortex ROI covering the calcarine sulcus was manually drawn on the high resolution T1 structural image in each hemisphere using the editable ROI function of MrDiffusion software (http://sirl.stanford.edu/software/). Probabilistic tractography was then employed between the LGN and calcarine ROIs using the ConTrack feature of MrDiffusion and parameters described by Sherbondy et al[10]. Initially, 70000 fibers were collected for OR tractography, of which the 30000 best fibers were selected by a scoring algorithm. OR fibers were then manually cleaned using Quench software (http://sirl.stanford.edu/software/). Meyer’s loop was clearly visible in all OR reconstructions.

### Lesion identification

**Fig 1:**
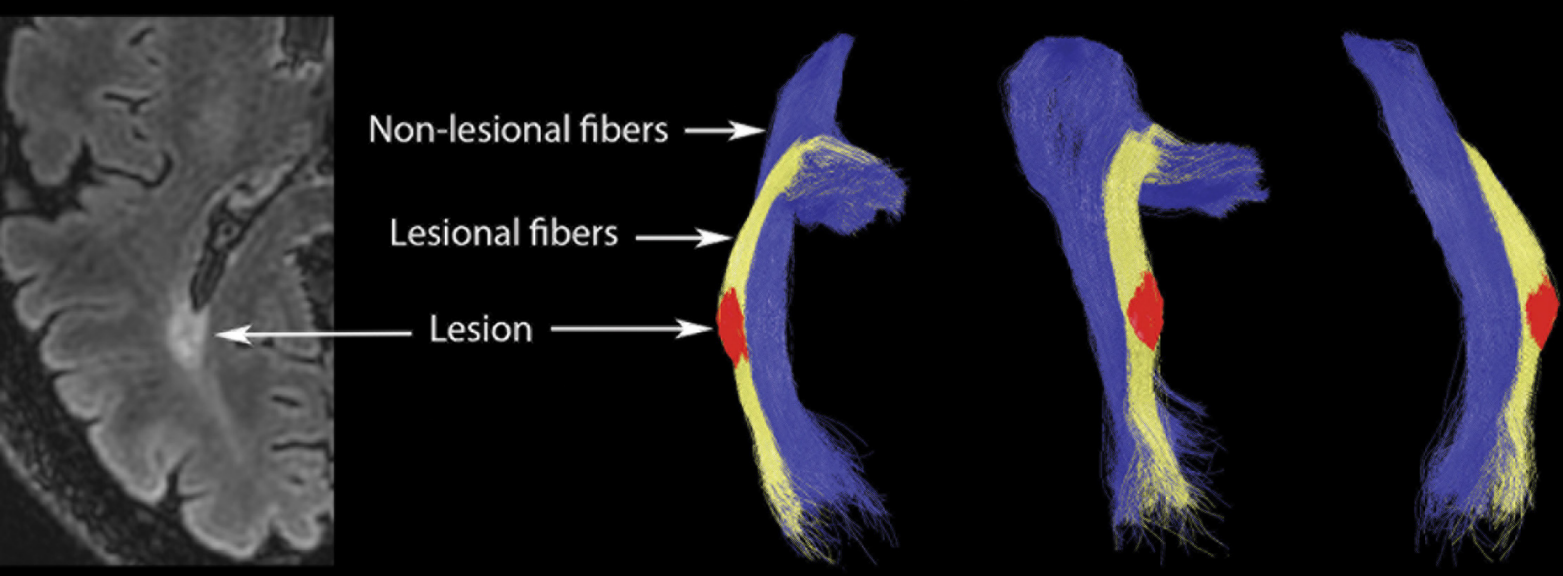
Optic radiation tractography and definition of OR lesions and non-lesional NAWM. Optic radiation was defined using probabilistic tractography. Optic radiation fibres were separated into lesional, i.e. traversing the lesion fibers (yellow), and non-lesional, i.e. non-traversing the lesion fibers (blue). Intersection between lesional fibers and brain lesion mask formed OR lesion ROI (red). Non-lesional fibers were transformed to non-lesional NAWM ROI.

MS lesions of the entire brain were identified on the co-registered T2 FLAIR and contrast-enhanced T1 images at baseline and follow up visits. Lesions were segmented automatically using ITK-SNAP 2 (ITK-SNAP, version 2, University of Pensilvania). Seed points were set by a single user (AK). Lesions were then intersected with OR fibers to identify and measure the volume of T2 FLAIR lesions within the OR. An OR lesion mask was then applied to baseline and follow-up DTI images and diffusivity was measured inside the mask using a voxel-based method (Fig 1).

### Non-lesional NAWM

Fibers traversing OR lesions can potentially be affected by retrograde and Wallerian degeneration (WD) caused by axonal transection. Therefore, in order to eliminate this factor, fibers not-traversing MS lesions (‘non-lesional’ fibres) were separated from the rest of the OR and converted to a ROI to identify non-lesional NAWM (Fig 1). Voxel-based diffusivity of non-lesional NAWM was then calculated for both baseline and follow-up scans.

For both lesions and non-lesional NAWM, extreme care was taken to remove voxels overlapping CSF. Considering potential atrophy-related CSF expansion during the follow-up period, the 12 month co-registered T1 image was used to manually remove CSF-contaminated voxels.

### Statistics

Statistical analysis was performed using SPSS 22.0 (SPSS, Chicago, IL, USA). Normality of data was tested using Shapiro-Wilk. Pearson correlation coefficient was used for bivariate correlation. Partial correlations was adjusted for age, gender, disease duration and history of ON. Student’s paired t-test was used to assess difference between baseline and follow-up diffusivity.

Univariate General Linear Model was used to analyse potential effect of various factors on lesional and non-lesional NAWM diffusivity change. Progression rate was used as a dependent variable.

Repeated measures General Linear Model was used to assess the effect of gender. Baseline and follow-up values of RD and AD were selected as within-subject variables, gender as a between-subject factor and brain and OR lesion load as covariates.

Where partial correlation was used, it was adjusted for age, sex, duration of the disease and history of ON.

## Results

Of 60 patients with RRMS, 39 demonstrated T2 FLAIR lesions within the OR at least on one side. Five patients developed new OR lesions (Gad or FLAIR) during follow-up period and were excluded from analysis. Therefore, data from 34 subjects (age: 44.8+/-10.2, disease duration: 4.9+/-3.6 y, 15M/19F, EDSS score: 1.42+/-1.38) were analysed. Sixteen patients (9F/7M) had history of ON more than 6 months prior to enrolment. Since the OR represents a paired structure, only one side was analysed. In patients who had lesions in both ORs (28 patients), one side was selected randomly. In six patients lesions occupied the entire cross-section of the OR; in such cases, there were no non-lesional fibres. Therefore, non-lesional NAWM was examined only in 28 ORs (11M/17F).

The average OR lesion volume was 608+/-575 mm^3^. Lesions varied in size considerably. In 50% of ORs with detectable lesions, lesional size was less than 500 mm^3^. Both RD and AD demonstrated much higher values within the lesions compared to non-lesional NAWM (p<0.001 for both) (Table 1).

**Table 1:** AD and RD values in T2 FLAIR lesions and non-lesional NAWM within the OR at baseline and follow-up visits.

	AD first visit	AD second visit	*p*	RD first visit	RD second visit	*p*
Lesions	1.58+/-0.16	1.61+/-0.16	**<0.001**	0.94+/-0.12	0.97+/-0.13	**<0.001**
males	1.57+/-0.14	1.62+/-0.15	**<0.001**	0.95+/-0.13	1.0+/-0.13	**<0.001**
females	1.58+/-0.18	1.59+/-0.18	**0.05**	0.94+/-0.13	0.95+/-0.13	**0.1**
NAWM	1.186+/-0.05	1.196+/-0.06	**0.005**	0.746+/-0.05	0.751+/-0.05	0.07
males	1.22+/-0.06	1.23+/-0.05	0.2	0.758+/-0.05	0.761+/-0.05	0.6
females	1.17+/-0.05	1.18+/-0.06	**0.01**	0.739+/-0.05	0.745+/-0.05	0.06

There was no correlation between baseline diffusivity indices (in both lesions and non-lesional NAWM) and whole brain lesion load or optic radiation lesion load (all correlations adjusted for age, gender, disease duration and history of ON, p>0.05 for all).

There was a statistically significant (p<0.001, paired t-test) increase of lesional AD and RD during the follow-up period (Table 1). AD also demonstrated a significant increase in non-lesional NAWM, albeit on a much lesser scale, while the change in RD was borderline. The largest relative increase was observed in lesional RD (2.5%), followed by lesional AD (1.8%), with non-lesional fibers showing 0.8% and 0.4% increase for AD and RD respectively. There was no correlation between EDSS score and diffusivity change for both lesional and non-lesional ROIs (P>0.05 for all).

**Fig 2:**
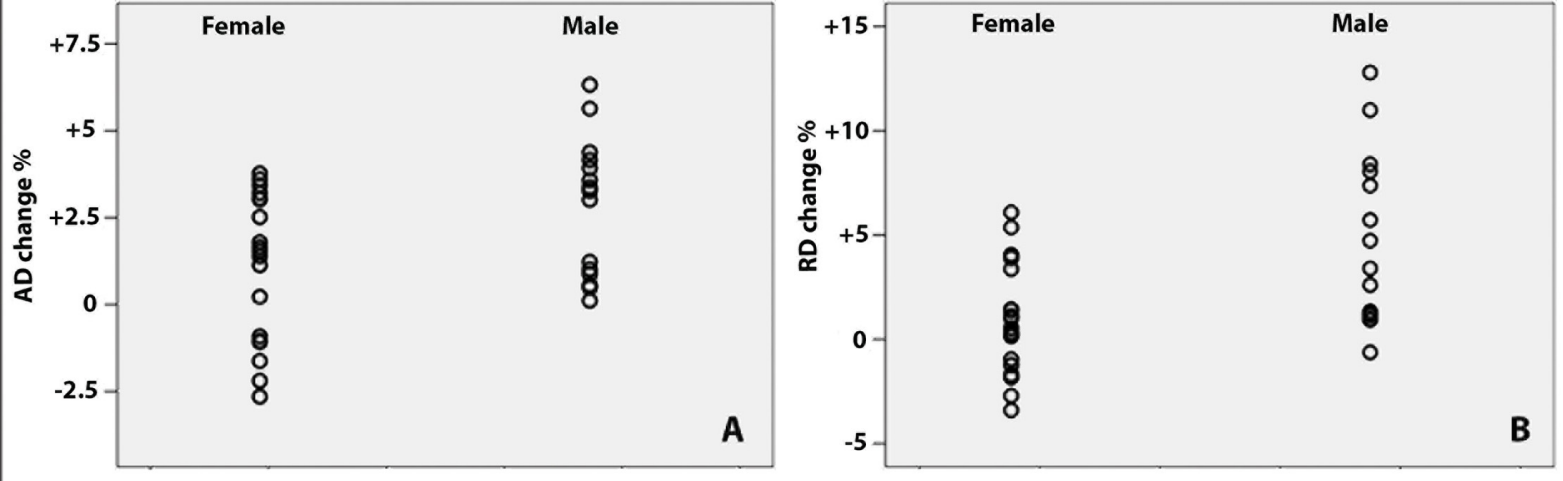
Relationship between gender and diffusivity progression. Regression standardized prediction plots of lesional diffusivity progression for gender groups. A-axial diffusivity, B-radial diffusivity.

A univariate linear regression model was used to assess the potential effect of various factors on diffusivity progression. Age, gender, disease duration, history of ON, OR lesion load and whole brain lesion load were entered into the model. While these variables showed no effect on diffusivity change in non-lesional NAWM, gender significantly impacted on progressive change of AD and RD in lesional tissue (Standardized b = 0.40 and b = 0.49, p = 0.02 and p = 0.003 for AD and RD respectively). Regression standardized prediction plots for gender groups are presented in Fig 2. No other variables demonstrated any effect.

The gender difference was confirmed using a Repeated Measures general linear model (GLM), when gender was selected as a between-subject factor. The model demonstrated significant contribution of gender to increase in lesional AD and RD (p = 0.019 and p = 0.003 respectively). None of the factors were significant for the non-lesional NAWM model.

While gender groups were balanced with respect to age and disease duration (p = 0.48 and p = 0.3 respectively) (Table 2), males demonstrated a larger lesion load. Therefore, both OR and total brain lesion volume were entered into the model as covariates to adjust for difference in lesion volume between the groups. This, however, did not alter the outcome of the model, which still demonstrated a significant gender effect on diffusivity progression (p = 0.019 and p = 0.001 for AD and RD respectively).

**Table 2:** Demographics, lesion volume and medications in male and female patients. Average +/-SD.

Gender	n	Age (years)	Disease duration (years)	Brain lesion load (mm3)	OR lesion load (mm3)	Medications
Female	19	45.8+/-12.2	4.4+/-3.3	3820+/-3099	467+/-529	None-1, Beta-interferon-7, Glatiramer acetate-8, fingolimod-3, natalizumab-0
Male	15	43.3+/-7.2	5.6+/-3.9	6800+/-5516	788+/-651	None-1, Beta-interferon-2, Glatiramer acetate-0, fingolimod-5, natalizumab-4, other-4

Partial correlation between OR lesion volume and AD or RD diffusivity change within lesions also did not show significance (p = 0.8 and p = 0.7 respectively), supporting the notion that lesion size *per se* does not influence diffusivity progression.

When lesions in males and females groups were analysed separately, the increase of AD in the female group demonstrated borderline significance (p = 0.05). Furthermore, change in the level of RD for females during the follow-up lost significance. In contradistinction, the difference between baseline and 12 months in the male group became larger for both AD and RD compared with the entire study population (Table 1). Thus, the female group demonstrated considerably smaller relative change during follow-up period for both lesional AD and RD (1.1% and 0.9% respectively) compared with the entire cohort (1.8% and 2.5% respectively), while progression rate for these indices increased to 2.8% and 4.6% in the male group. This difference in relative progression of lesional diffusivity was significant between the gender groups (p = 0.019 and p = 0.003 for AD and RD respectively).

## Discussion

Longitudinal assessment of DTI metrics in patients with MS has been studied in spinal cord, whole brain tissue, normal appearing white and grey matter (NAWM, NAGM) and acute and chronic lesions[11][12]. Typically, however, only composite DTI indices, such as Fractional Anisotropy (FA) and Mean Diffusivity (MD) were used or combined analysis of lesional and NAWM performed.

As such, the current study represents the first attempt to analyse parallel and perpendicular diffusivity changes within MS lesions of a single white matter tract. In addition, non-lesional NAWM analyzed in this study is represented by OR fibers that do not transect lesions at any point and are, therefore, not affected by anterograde and Wallerian degeneration.

### Diffusivity changes in MS lesion

We have demonstrated progressive microstructural changes in chronic MS lesions that can be detected *in vivo* at a relatively early stage of the disease using DTI. The pathological substrates of such changes are yet to be fully delineated. In normal fibres, RD is likely restricted by both the axonal membrane and the myelin sheath. While perpendicular diffusivity is considerably lower than diffusivity parallel to the fibers, demyelination can significantly “modulate” this relationship[3], prompting speculation that RD may potentially be used as a marker of myelination[6][13]. In concordance with this hypothesis, we recently demonstrated that a marked, focal increase of RD is topographically linked with chronic T2 OR lesions, and potentially relates to the degree of lesional myelin loss[8]. Therefore, a progressive increase in lesional RD over time, as demonstrated in the current study, suggests the presence of continuous demyelination in chronic MS lesions.

Altered AD, on the other hand, has been frequently attributed to axonal loss. Contrary to animal models of Experimental Autoimmune Encephalomyelitis, however, studies of human brain in MS patients typically revealed elevated level of AD[14][15]. Our recent study has also demonstrated a significant increase of AD in OR MS lesions with ‘spread’ to the segment of the axon distal to the injury^8^. This pattern of change is consistent with axonal transection within lesions and subsequent distal axonal loss due to Wallerian degeneration. Therefore, the gradual increase of AD in individual chronic MS lesions over time, described here, may indicate progressive axonal damage, possibly due to low grade inflammation and/or vulnerability of demyelinated axons.

Based on these observations, it is likely that progressive microstructural alteration detected within lesional OR tissue in this study is related to ongoing inflammatory demyelination and axonal damage. This is supported by recent studies demonstrating “slow burning” inflammation and lesion expansion in chronic MS lesions[16][17], which may be linked to secondary demyelination of incompletely remyelinated axons[18] and consequent axonal loss, particularly at the lesion border[19]. Such slow continuous inflammatory demyelination and axonal injury in pre-existing lesions may “represent a pathologic substrate of gradual worsening of pre-existing clinical deficits”[2]. This relatively indolent process may be masked during the relapsing-remitting stage of MS by both the acute clinical features associated with the development of new focal lesions and the remarkable compensatory ability of the brain in early course of the disease, but becomes increasingly apparent in the progressive stage of the disease.

Another important finding of the current study is the gender-specific difference in the progressive change of lesional diffusivity. The univariate linear regression model, which was used to assess potential effect of various factors on diffusivity progression, demonstrated that, within lesions, gender was the only factor producing a significant effect on change of both AD and RD. Specifically, the increase in both parallel and perpendicular diffusivity during the 12 months follow-up period in our cohort was mostly driven by the male sub-group, while females demonstrated much smaller changes in lesional diffusivity. The F:M progression rate for RD, for example, reached 1:6.5.

Despite the fact that the OR lesion load in the male group was twice as large as in the female group, lesion volume (both brain and OR) did not contribute significantly to the univariate linear regression model. Repeated Measures GLM with gender as the between-subject factor also confirmed significant contribution of sex to both AD and RD change, which was not altered when OR and total brain lesion volume were entered into the GLM as covariates.

Together with the absence of a significant correlation between OR lesion volume and diffusivity progression, this suggests that gender, rather than lesion size, is responsible for the sex-related difference in diffusivity progression.

This dissimilarity in the rate of microstructural change in chronic MS lesions may reflect a pathophysiological difference between males and females in relation to the disease evolution. While the incidence of MS is consistently greater in females[20], males have been reported to have a more severe disease phenotype resulting in a worse clinical outcome and faster accumulation of disability[21][22]. A recent registry-based study, including data from more than 14000 patients, demonstrated significantly faster disease progression in males with relapse-onset disease[23]. Gender, however, does not appear to influence relapse rate[24] or the burden of cerebral T2 lesions[25].

The pathophysiological basis of this gender disparity in MS is poorly understood. Female sex hormones may confer a higher susceptibility to autoimmunity (see[26] for review); and experimental allergic encephalomyelitis (EAE) is more readily induced in female mice (which develop a pronounced Th1 pro-inflammatory cytokine response) or by female T lymphocytes (see[27] for review). Conversely, male hormones appear to protect against the development of autoimmunity; and a Th2 (anti-inflammatory) type cytokine response predominates in experimental male animals[28].

Gender-related factors may also play a role in disease progression. Several studies have shown a protective role of female sex hormones, and the pregnancy hormone estriol in particular, on MS course (see[29] for review). Estriol reduces inflammation and promotes axon and myelin survival, which may contribute to the beneficial role of pregnancy on MS. Increased synthesis of progesterone, which has both neuroprotective and pro-myelinating effects on the CNS[21], has also recently been reported to be specific to female, but not male MS lesions[30]. The male sex hormone testosterone, on another hand, was shown to amplify excitotoxic damage to oligodendrocytes and may potentially limit remyelination[31].

In addition, sexual dimorphism in brain structure and the ability to repair injury and effect remyelination; direct effects of the X chromosome; and gender-specific epigenetic variation and gene-environment interactions may differentially impact MS prevalence and disease course (see[32] for review).

### Diffusivity changes in non-lesional NAWM

Non-lesional NAWM demonstrated significantly lower diffusivity metrics (both AD and RD) at the onset of the study and a much slower progression rate compared to lesions. There was also no gender bias in non-lesional NAWM with respect to diffusivity change.

The large volume of NAWM relative to lesional white matter suggests that even modest changes in the former may contribute significantly to the disease progression[33]. Accordingly, NAWM tissue damage, assessed using advanced MRI techniques, has been reported in multiple studies (see[34] for review) and has been predominantly attributed to axonal loss subsequent to WD[33]. However, since only non-lesional fibers were selected to create the non-lesional NAWM ROI in the current study, it is unlikely that WD had a direct effect on longitudinal microstructural changes in non-lesional NAWM[8]. This is also supported by the absence of a correlation between diffusivity indices and OR lesion load.

While several pathological processes, including axonopathy, trans-synaptic degeneration or microscopic lesions beyond the resolution of the current MRI technique may contribute to the progressive diffusivity change observed in non-lesional NAWM, the study design does not permit the delineation of a specific pathological mechanism.

The interaction between gender and brain damage may influence the clinical responses to disease modifying treatment[35]. However, in the context of a relatively small sample size, it was not feasible to assess the potential impact of disease modifying therapies on our results. Female patients in our cohort were more likely to be receiving conventional treatments (beta-interferon and glatiramer acetate), while the majority of males were receiving more aggressive therapy with fingolimod or natalizumab. While this fact by itself is consistent with more severe disease in males, it also suggests that DMT does not substantially influence diffusivity progression, since highly efficacious therapies should theoretically better prevent progression of the disease.

The lack of longitudinal DTI data in normal controls represents a limitation of our study. However, previous investigation of white matter microstructure using DTI has found no difference between sexes[36]. A recent study of age-related changes of AD and RD in normal subjects revealed that increases in diffusivity occurred in a more widespread fashion in females. In both sexes, however, diffusivity of occipital brain remains stable[37], supporting, therefore, the disease-related nature of changes observed in the current study.

## Conclusion

The significant increase of both axial and radial diffusivity in chronic MS lesions demonstrated in this study suggests ongoing inflammatory and demyelinating activity accompanied by axonal loss. In addition, the findings of the current study support the recently observed trend of more rapid disease progression in males and establish DTI as a potential *in vivo* biomarker of this dichotomy by demonstrating a significantly faster rate of microstructural change in the chronic lesions of male patients with MS.
